# Horse Meat Hydrolysate Ameliorates Dexamethasone-Induced Muscle Atrophy in C57BL/6 Mice via the AKT/FoxO3a/mTOR Pathway

**DOI:** 10.3390/cells14141050

**Published:** 2025-07-09

**Authors:** Hee-Jeong Lee, Dongwook Kim, Yousung Jung, Soomin Oh, Cho Hee Kim, Aera Jang

**Affiliations:** 1 Department of Applied Animal Science, Kangwon National University, Chuncheon 24341, Republic of Korea; doob2029@naver.com (H.-J.L.); donguk8282@naver.com (D.K.); dbtjd97@naver.com (Y.J.); osm808@naver.com (S.O.); 2 College of Nursing, Kangwon National University, Chuncheon 24341, Republic of Korea; chkim@kangwon.ac.kr

**Keywords:** horse meat, muscle atrophy, hydrolysate, sarcopenia, dexamethasone, proteolysis

## Abstract

As life expectancy increases, muscle atrophy, characterized by a decline in muscle mass and strength that can impair mobility, has become a growing concern, highlighting the potential of protein supplementation as a promising intervention strategy. A horse meat hydrolysate, with a molecular weight of less than 3 kDa, derived from m. *biceps femoris* and produced using the food-grade enzyme Alcalase^®^ (A4 < 3kDa) was evaluated for its efficacy in mitigating dexamethasone-induced muscle atrophy, a widely accepted model for studying catabolic muscle loss. Administered orally to C57BL/6 mice at dosages of 200 mg/kg or 500 mg/kg body weight for 35 days, A4 < 3kDa effectively countered the weight loss induced by dexamethasone in the whole body, quadriceps, tibialis anterior, and gastrocnemius muscles. Moreover, it increased muscle fiber cross-sectional area and grip strength. These effects were attributed to increased protein synthesis via the protein kinase B (AKT)/forkhead box O3 (FoxO3a)/mammalian target of rapamycin (mTOR) signaling pathway. A4 < 3kDa augmented the phosphorylation of key components of the signaling pathways associated with muscle atrophy, resulting in reduced mRNA expression of Atrogin-1 and MuRF-1. These findings demonstrate the potential of A4 < 3kDa as a functional food ingredient for preventing muscle atrophy.

## 1. Introduction

Skeletal muscle, accounting for 40% of total body weight, plays a pivotal role in basal energy metabolism and heat generation to maintain body temperature. Additionally, it converts chemical energy into mechanical movement, facilitating functions such as force generation, postural maintenance, and various activities. As fertility declines and the global population ages, skeletal muscle atrophy, a hallmark of aging, has gained increased importance [1,2]. This condition can lead to diminished life quality, greater physical disability, and even increased mortality.

Sarcopenia, or age-related muscle atrophy, is a recognized muscle disease with an ICD-10-MC diagnosis code used for clinical billing in some countries. The term originates from the Greek words sarx (flesh) and penia (loss) [3]. In 2010, the European Working Group on Sarcopenia in Older People (EWGSOP) proposed a widely adopted definition, characterizing sarcopenia as the progressive loss of skeletal muscle mass with aging. However, a universally accepted definition applicable to both research and clinical practice remained lacking. To address this, EWGSOP revised the criteria in 2018, defining sarcopenia as a muscle failure syndrome resulting from lifelong adverse muscle changes. Although more prevalent in older adults, it can also occur earlier in life. The updated definition is based on three diagnostic parameters: (1) low muscle strength, (2) reduced muscle quantity or quality, and (3) poor physical performance, the latter serving as an indicator of severity [4].

Sarcopenia involves multiple aspects. Firstly, there is a notable reduction in the cross-sectional area (CSA) of muscle fibers, which has been extensively documented and is often linked to decreases in both muscle mass and strength [5]. Secondly, a reduction in the number of muscle fibers is observed. Skeletal muscle fibers are differentiated into two primary types, slow-twitch (type I) and fast-twitch (type II) fibers, which are associated with endurance activities and high-intensity performance, respectively [6]. It is important to note that the declines are more pronounced in type II fibers, which can act as predictors of muscle strength and significantly contribute to the loss of muscle strength [7,8]. These symptoms can be attributed to elevated glucocorticoids’ levels associated with the aging process [9,10]. While dexamethasone-induced muscle atrophy does not entirely replicate the chronic and multifactorial nature of age-related sarcopenia, it has been widely utilized as an established experimental model due to its ability to reliably induce rapid skeletal muscle loss.

To attenuate sarcopenia, dietary protein supplementation has emerged as a viable strategy, especially given that older individuals often fall short of the recommended average protein intake of 0.7 g/kg body weight (BW)/day [11,12,13]. Among protein-rich options, meat stands out as a highly nutritious and energy-dense natural food product, containing essential amino acids in abundance [14,15]. Notably, horse meat is distinguished by its impressive protein content, reaching up to 24.5%. The composition of its essential amino acids, which are particularly effective in stimulating muscle protein synthesis compared to nonessential amino acids, makes up 54.5% of its protein content, exceeding that of beef, which contains 47.4% [16,17]. Despite these nutritional benefits, horse meat is often noted for its tough texture. Enzymatic hydrolysis can mitigate this issue by breaking down myofibrillar proteins and thus improving tenderness [18]. Importantly, this process not only enhances the protein quality but also adds functional properties, such as anti-inflammatory and antioxidant effects [19,20].

In our previous research, we found that horse meat hydrolysate, specifically A4 < 3kDa, led to the increased phosphorylation of AKT, mTOR, and FoxO3a, along with a reduction in mRNA expression of Atrogin-1 and MuRF-1. These effects were linked to its antioxidant properties, evidenced by improved cell viability in H_2_O_2_-afflicted C2C12 myoblast cells and the decreased production of reactive oxygen species. Additionally, A4 < 3kDa demonstrated anti-inflammatory properties by lowering the mRNA levels of cytokines such as IL-6 and TNF-α in C2C12 muscle cells [21].

To confirm if A4 < 3kDa could reproduce these effects in vivo, this study sought to assess its impact on dexamethasone-induced muscle atrophy in a C57BL/6 mouse model, evaluating parameters such as muscle weight, muscle fiber CSA, and investigating molecular mechanisms related to signaling pathways involved in dexamethasone-induced muscle atrophy.

## 2. Materials and Methods

### 2.1. Preparation of Low-Molecular-Weight Horse Meat Enzyme Hydrolysates (A4 < 3kDa)

Horse meat enzyme hydrolysate was prepared following the method described by Lee et al. [21]. In brief, horse meat (m. *biceps femoris*) was combined with distilled water at a 1:5 ratio. Subsequently, 1% (*v*/*v*) Alcalase^®^ (Novozymes, Bagsvaerd, Denmark), a food-grade enzyme, was added, and hydrolysis was carried out for 4 h at an optimal temperature of 50 °C and a pH of 8.0. After the enzymatic reaction, the enzyme was deactivated by heating the solution to 100 °C for 10 min. The hydrolysate was then filtered using an Amicon Ultra centrifugation filter (Merck Millipore, Billerica, MA, USA) to segregate hydrolysates below 3 kDa (A4 < 3kDa). The solution was subsequently lyophilized and stored at −20 °C until further analysis.

### 2.2. Animals and Treatment

**Figure 1:**
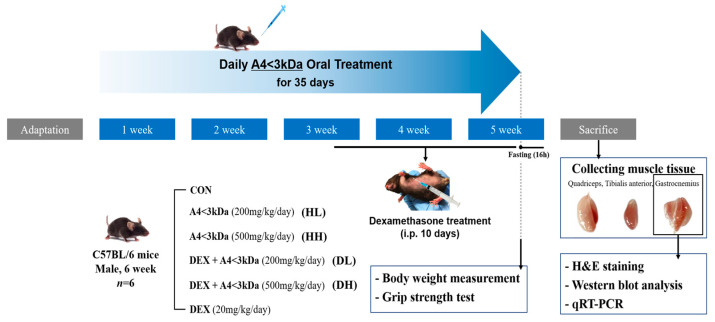
Animal experiment design. CON = normal group; DEX = dexamethasone-treated group; DL = A4 < 3kDa (200 mg/kg BW)-administrated and dexamethasone-treated group; DH = A4 < 3kDa (500 mg/kg BW)-administrated and dexamethasone-treated group; HL = A4 < 3kDa (200 mg/kg BW)-administrated group; HH = A4 < 3kDa (500 mg/kg BW)-administrated group.

Four-week-old male C57BL/6 mice were acquired from DBL (Eumseong, Republic of Korea) and maintained under controlled conditions (23 ± 2 °C, 12 h:12 h light–dark cycle) with unrestricted access to water and food (Teklad Global 18% Protein Rodent Diet, ENVIGO, Indianapolis, IN, USA). After a 7-day acclimation period, the mice were randomly assigned to one of six groups (n = 6 per group). The control group (CON) received an oral administration of 0.2 mL of tap water daily for 35 days, while a polyethylene glycol (PEG) 400 solution (30% PEG 400 in 0.9% saline) was administered intraperitoneally for the last 10 days. The dexamethasone-only treated group (DEX) received injections of tap water and was treated with 20 mg/kg BW of dexamethasone in a PEG 400 solution. A4 < 3kDa at 200 mg/kg BW or 500 mg/kg BW was diluted in tap water and combined with the dexamethasone treatment (for groups DL and DH, respectively). Without the dexamethasone treatment, two concentrations of A4 < 3kDa were administered (HL for low concentration and HH for high concentration). Prior to administration, all solutions were sterilized through a 0.22 µm membrane filter (ADVANTEC, Tokyo, Japan). In short, after a 7-day acclimation period, animals received daily oral administration of either A4 < 3kDa or tap water from day 8 to day 42 (10:00–11:00 a.m.). Intraperitoneal injections of dexamethasone or the PEG 400 solution were administered from day 33 to day 42 (4:00–5:00 p.m.). Body weights were measured weekly throughout the study. Following a 16 h fasting period, the mice were euthanized by cardiac puncture under anesthesia, and the gastrocnemius (GA), tibialis anterior (TA), and quadriceps muscles (QD) were harvested and then preserved at −80 °C. All animal experiments adhered to the ethical guidelines of Kangwon National University (KW-230413-1, approved on 6 July 2023). The experimental design is depicted in Figure 1.

### 2.3. Measurement of Muscle Mass and Grip Strength of C57BL/6 Mice

The body weight of mice was recorded weekly throughout the experimental period. Muscle function was evaluated by measuring grip strength using a grip strength meter (Bioseb, Chaville, France) at the final time point. The mice grasped the T bar with their forelimbs, and their tails were pulled back horizontally until they released the bar. Each mouse underwent the test three times, and the average force values were computed. The grip strength values were presented in grams.

### 2.4. Histological Analysis

The isolated GA muscle tissues were fixed in 4% paraformaldehyde, then embedded in paraffin and sectioned into 4 µm slices. These sections were stained with hematoxylin and eosin (H&E) and inspected under a microscope (×200). For the CSA analysis, 6 images per group were taken, and 20 myofibers per image were randomly assessed. CSA measurements were conducted using ImageJ software 1.54m version (National Institute of Health, Bethesda, MD, USA).

### 2.5. Protein Extraction and Western Blot Analysis

The isolated GA muscle was homogenized in easy-BLUE™ (iNtRON, Seongnam, Republic of Korea), supplemented with a protease inhibitor cocktail (Sigma-Aldrich, St. Louis, MO, USA). The lysate was then incubated at −20 °C for 30 min before being centrifuged at 15,928× *g* for 10 min at 4 °C. The supernatants were collected for a Western blot analysis. Protein concentrations were determined using a BCA protein assay kit (Thermo Scientific, Rockford, IL, USA). An equal amount of protein (35 µm) was loaded onto a 6–10% SDS-PAGE gel and transferred onto a polyvinylidene fluoride (PVDF) membrane. The membrane was blocked with 5% bovine serum albumin (Bovogen, Melbourne, Victoria Australia) at 25 ± 1 °C for 2 h and incubated overnight at 4 °C with primary antibodies against AKT (1:2000; #2920, Cell Signaling Technology, Danvers, MA, USA), FoxO3a (1:1000; #2497, Cell Signaling Technology), mTOR (1:1000; #2983, Cell Signaling Technology), ribosomal protein S6 kinase beta-1 (p70S6K) (1:1000; #9202, Cell Signaling Technology), eukaryotic translation initiation factor 4E-binding protein 1 (4EBP1) (1:1000; #9452, Cell Signaling Technology), *p*-AKT (1:1000; #4051, Cell Signaling Technology), *p*-FoxO3a (1:1000; #13129, Cell Signaling Technology), *p*-mTOR (1:1000; #2971, Cell Signaling Technology), *p*-p70S6K (1:1000; #9205, Cell Signaling Technology), and *p*-4EBP1 (1:1000; #2855, Cell Signaling Technology). Following 0.05% TBST (25 mM Tris, 0.05% Tween 20, and 140 mM NaCl) washes, the membranes were probed with anti-mouse or anti-rabbit IgG antibodies at room temperature. Protein bands were visualized using enhanced chemiluminescence (Cyanagen, Bologna, Italy) and the expression level was analyzed by detecting the density of the band using Fusion Solo 6S Edge (Vilber-Lourmat, Marne la Vallée, France).

### 2.6. RNA Extraction and Quantitative Real-Time Polymerase Chain Reaction (qRT-PCR) Analysis

The GA muscle tissues were isolated, homogenized using easy-BLUE™ (iNtRON, Republic of Korea), and total RNA was extracted with an RNeasy Mini kit (Qiagen, Hilden, Germany) following the manufacturer’s guidelines. cDNA was synthesized with Pre-Mix (dT18 Plus) (BioFact, Daejeon, Republic of Korea) and subsequently amplified using the LightCycler 96 System (Roche Diagnostics, Basel, Switzerland) alongside 2X Real-Time PCR Master Mix (BioFact). β-actin served as the reference gene to normalize mRNA levels. Table 1 lists the primers and the corresponding annealing temperatures used for the amplification process.

### 2.7. Statistical Analysis

Statistical analyses were conducted using the Statistical Analysis System (SAS) program v.9.4, which involved an analysis of variance (ANOVA). Each experiment was independently repeated three times, yielding three distinct data sets for each condition. Results are presented as means ± standard deviation, derived from these replicates. A *p*-value of <0.05 was used to define statistical significance, adopting Tukey’s test for multiple comparisons.

## 3. Results and Discussion

### 3.1. Body and Muscle Weight in Dexamethasone-Induced Muscle Atrophy in Mice

To determine the mitigating effect of A4 < 3kDa on dexamethasone-induced muscle atrophy in C57BL/6 mice, body weight (BW) was recorded biweekly. Initial weight differences were not significant until the 21st day of the experiment (Table 2). However, following the administration of dexamethasone, the BW of the DEX group (21.90 g) was significantly reduced compared to the CON group (23.68 g) on the 28th day (*p* < 0.05). Treatment with A4 < 3kDa in both DL and DH groups (showing 23.00 g and 22.97 g BW, respectively, on the 28th day) helped counteract this decline, aligning weights close to those of the CON group. Moreover, the HL and HH groups also showed similar weights to the CON group. By the 35th day, the BW in the DL and DH groups had increased compared to the DEX group. While these increases did not match those seen in the CON group, an alleviation of BW loss due to dexamethasone was observed with the A4 < 3kDa treatment (Table 2). Jeon et al. assessed the impact of administering a hot water extract of antler (NFA) or *Lactobacillus curvatus* fermented antler (FA) to C57BL/6 mice also treated with dexamethasone [22]. They found that while BW loss commenced from the 14th day in the dexamethasone-groups, administering 120 mg/kg BW of NFA and FA for 24 days did not significantly increase the body weight when compared to the dexamethasone-only treated group.

**Figure 2:**
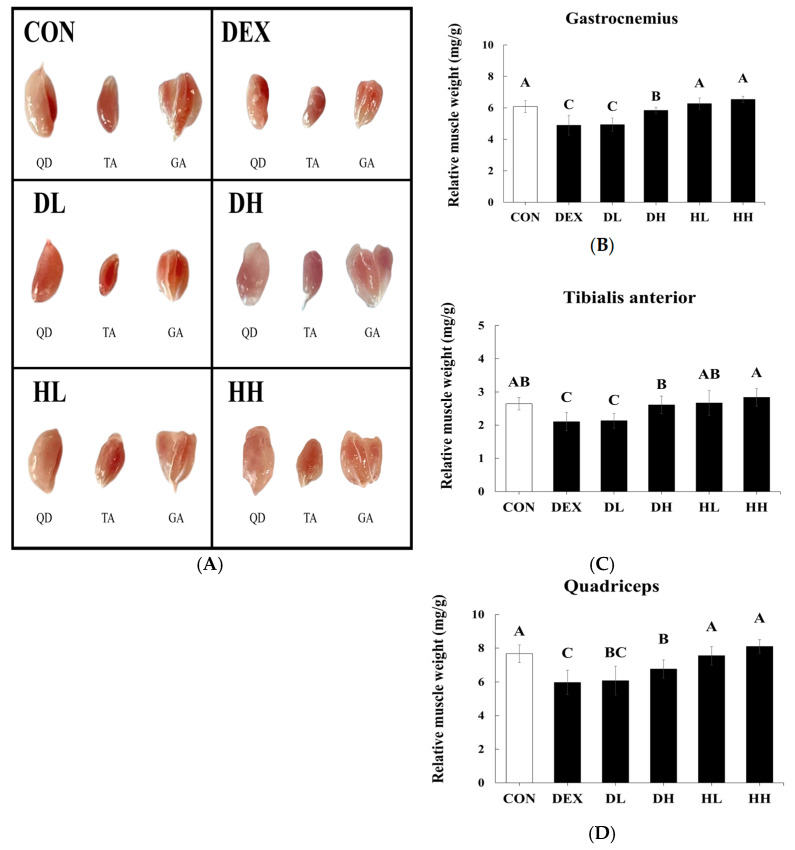
Effect of A4 < 3kDa on QD, TA, and GA muscles isolated from C57BL/6 mice. (**A**) Isolated QD, TA, and GA muscles from mice. (**B**) Relative weight of GA muscle. (**C**) Relative weight of TA muscle. (**D**) Relative weight of QD muscle. CON = normal group; DEX = dexamethasone-treated group; DL = A4 < 3kDa (200 mg/kg BW)-administrated and dexamethasone-treated group; DH = A4 < 3kDa (500 mg/kg BW)-administrated and dexamethasone-treated group; HL = A4 < 3kDa (200 mg/kg BW)-administrated group; HH = A4 < 3kDa (500 mg/kg BW)-administrated group; QD = quadriceps; TA = tibialis anterior; GA = gastrocnemius. ^A–C^ Values of bar with different superscripts among treatments differ significantly at *p* < 0.05.

To determine changes in three types of muscle tissues, the TA, GA, and QD were collected (Figure 2A). Given that TA, GA, and QD muscles primarily consist of fast-twitch fibers (type II) and elevated levels of glucocorticoids such as treatment with dexamethasone and symptoms of the aging process preferentially affect type II muscle fibers, we measured the weight of TA, GA, and QD muscle tissues (Figure 2B–D) [23,24,25].

Upon administration of dexamethasone, all muscle tissues showed a significant weight decrease compared to the CON group (*p* < 0.05), mediated by treating with 500 mg/kg BW of A4 < 3kDa. The weight of QD, TA, and GA muscles in the DH group increased by 13.40%, 23.70%, and 19.98%, respectively, compared to the DEX group. Additionally, in the HL and HH groups, A4 < 3kDa caused a slight increase in the weight of three muscle tissues compared to the CON group. A4 < 3kDa enhanced the weight of QD, TA, and GA muscles by 5.61%, 7.17%, and 7.57%, respectively, compared to the CON group, although these differences were not statistically significant.

These results are consistent with previous studies investigating extracts or hydrolysates from various natural sources. Seo et al., who examined the effect of *Psoralea corylifolia* L. seed extract on dexamethasone treated C57BL/6 mice, reported that the extract increased QD muscle by approximately 18.18%, TA muscle by 16.67%, and GA muscle by 20% at a concentration of 500 mg/kg of the extract [10]. Furthermore, Jeon and Choung discovered that oyster hydrolysate, processed with Protamex^®^ and Neutrase^®^, increased the weight of dexamethasone-inflicted GA and QD muscles by 13.95% and 12.50%, respectively, at a dosage of 200 mg/kg BW [26].

### 3.2. CSA of Muscle Fibers in Mice with Dexamethasone-Induced Muscle Atrophy

Glucocorticoids are known for inducing morphological alterations in muscle-atrophy conditions, including reduced muscle fiber size, loosen arrangement, and expanded interfiber spaces [27]. Notably, the GA muscles, which comprise a significant portion of the hindlimb, have the capacity for high force generation [26]. Therefore, we focused on morphological observations using the GA muscle.

**Figure 3:**
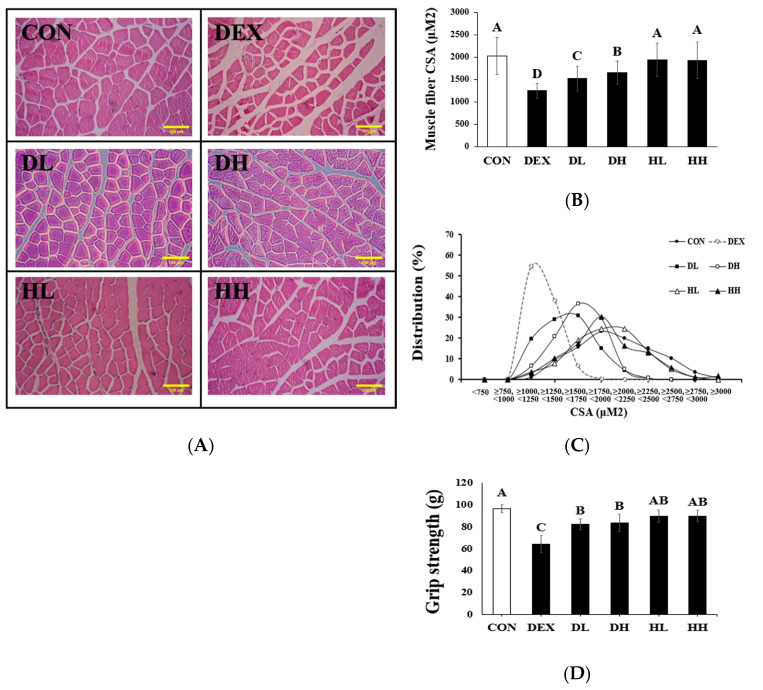
Effect of A4 < 3kDa on the structural damage of muscle tissue and muscle function in mice with dexamethasone-treated muscle atrophy. (**A**) Histological morphology of GA muscle in mice. (**B**) CSA of 120 randomly selected muscle fibers. (**C**) Muscle fiber CSA distribution. (**D**) Grip strength of the forelimbs in mice. CON = normal group; DEX = dexamethasone-treated group; DL = A4 < 3kDa (200 mg/kg BW)-administrated and dexamethasone-treated group; DH = A4 < 3kDa (500 mg/kg BW)-administrated and dexamethasone-treated group; HL = A4 < 3kDa (200 mg/kg BW)-administrated group; HH = A4 < 3kDa (500 mg/kg BW)-administrated group; ^A–D^ Values of bar with different superscripts among treatments differ significantly at *p* < 0.05.

Figure 3A illustrates sections of GA muscle stained with H&E. Compared to the CON group, the DEX group exhibited an enlarged interfibrous area and a reduction in myofiber size. These changes improved following the administration of A4 < 3kDa to dexamethasone-treated mice. Treatment with dexamethasone resulted in a 38.37% decrease in fiber CSA in the DEX group compared to the CON group. Furthermore, when comparing the CSA in the DEX group to the DL and DH groups, A4 < 3kDa significantly increased CSA by up to 24.32% (*p* < 0.05, Figure 3B). Additionally, the CSA distribution graph demonstrated that the DEX group exhibited a left-leaning distribution, indicating a predominance of smaller CSA sizes (750–1500 μm^2^). In contrast, the distribution in the CON group appeared relatively even on the right side and concentrated between 1750 and 3000 μm^2^. In comparing the DEX-treated group with the combined A4 < 3kDa group (DL and DH), it was notable that 51.25% and 72.08% of fibers in the respective groups were within the 1500–2500 μm^2^ range, whereas the DEX group had only 7.08% of fibers in that range. The HL and HH groups exhibited a wide distribution between 1750 and 2250 μm^2^, similar to the distribution observed in the CON group (Figure 3C). These increases in CSA corresponded with a concurrent rise in GA muscle weight.

Oh et al. observed an increase of approximately 66.7% in muscle fiber CSA in the GA muscle, which reached a level comparable to that of the control group [28]. This enhancement was noted following treatment with a 3kDa collagen peptide primarily composed of glycine, hydroxyproline, and glutamic acid. Similarly, Wang et al. reported a 13.56% increase in muscle fiber CSA in 8-week-old male C57BL/6 mice subjected to 10 days of dexamethasone-induced muscle atrophy, following a 10-day oral administration of monotropein at a dose of 80 mg/kg body weight [27]. Monotropein, the prevalent compound in *Morinda officinalis* root, also demonstrated an anti-inflammatory effect. Likewise, Kim et al. conducted a study on a hydrolysate derived from horse, which reported that horse leg bone hydrolysate, produced through enzymatic degradation using pepsin and pancreatin, displayed superior oxygen radical absorbance capacity effects compared to pork skin extract [29]. Furthermore, our earlier investigation demonstrated that A4 < 3kDa exhibited antioxidant effects by reducing reactive oxygen species production and enhanced cell viability against H_2_O_2_-induced cellular toxicity in C2C12 muscle cells [21]. CSA, a robust indicator of muscle size, also correlates with the capacity to generate force in healthy individuals [30]. In investigating the effect of A4 < 3kDa on dexamethasone-induced muscle function, grip strength was measured on the last day of the experiment. The administration of dexamethasone decreased grip strength in mice by up to 33.54% compared to the CON group; this reduction was mitigated by 28.38% and 29.95% after administering A4 < 3kDa at doses of 200 and 500 mg/kg BW, respectively (Figure 3D).

Seo et al. reported that administering 500 mg/kg of *Psoralea corylifolia* L. seed extract significantly enhanced grip strength by approximately 25% in 8-week-old mice treated with dexamethasone for 12 days [10]. Furthermore, Jeon et al. discovered that a dosage of 120 mg/kg BW of FA over 24 days resulted in a notable 42.86% increase in grip strength in dexamethasone-treated mice, similar to that observed in the control group [22]. They attributed this improvement to an increased sialic acid content due to fermentation. Sialic acid deficiency, which arises under conditions of muscle damage, can lead to oxidative stress and muscle weakening [31,32]. Moreover, free sialic acid has been demonstrated to mitigate oxidative stress by neutralizing harmful H_2_O_2_ under physiological conditions [33]. Notably, horse meat has been identified as having a 1.60 times higher content of the free 2-keto-3-deoxy-D-glycero-D-galacto-nononic acid, a form of sialic acid, compared to pork, suggesting a potential mechanism by which A4 < 3kDa could counter muscle atrophy by alleviating oxidative stress induced by dexamethasone [34,35]. However, the precise amount of sialic acid present in A4 < 3kDa has not been determined, requiring the need for further detailed investigations.

### 3.3. Effect of A4 < 3kDa on Proteins Through the AKT/mTOR/FoxO3a Pathway in Mice Experiencing Dexamethasone-Induced Muscle Atrophy

**Figure 4:**
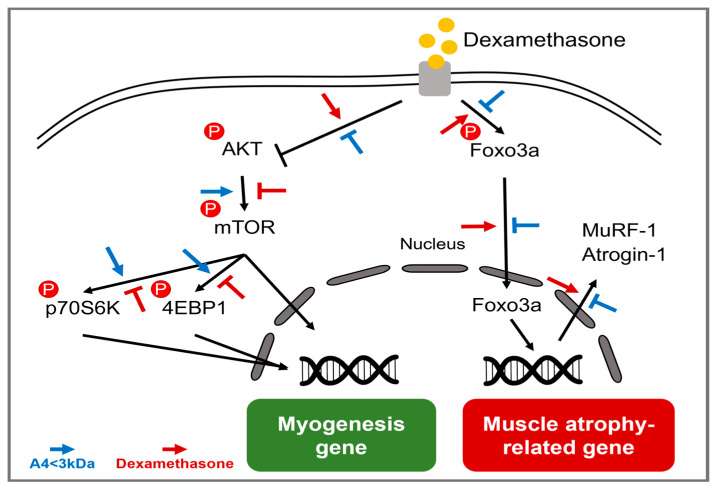
Summarized molecular mechanisms of muscle atrophy induced by glucocorticoid.

To elucidate the molecular mechanisms underlying the observed increases in the weight of muscle tissues and the CSA of muscle fibers, we examined the expression levels of signaling pathways related to muscle atrophy. Figure 4 illustrates the signaling pathways associated with the dexamethasone-induced ubiquitin–proteasome system. The proteolytic effects of dexamethasone primarily involve the ubiquitin–proteasome system, featuring muscle-specific E3 ubiquitin ligases such as Atrogin-1 and MuRF-1, which are crucial for protein ubiquitination and degradation [36]. Concurrently, FoxO3a, which is known to promote autophagy in skeletal muscle cells, increases the transcription of Atrogin-1 and MuRF-1. Meanwhile, the AKT/mTOR pathway is recognized as a critical regulator of protein synthesis and muscle hypertrophy [37]. Activated AKT enhances protein synthesis by upregulating mTOR, which subsequently phosphorylates downstream proteins such as p70S6K and 4EBP1. Additionally, activated AKT suppresses protein degradation by inhibiting FoxO3a [38]. Therefore, promoting AKT/mTOR signaling and reducing FoxO3a activation can be strategic in preventing muscle atrophy.

**Figure 5:**
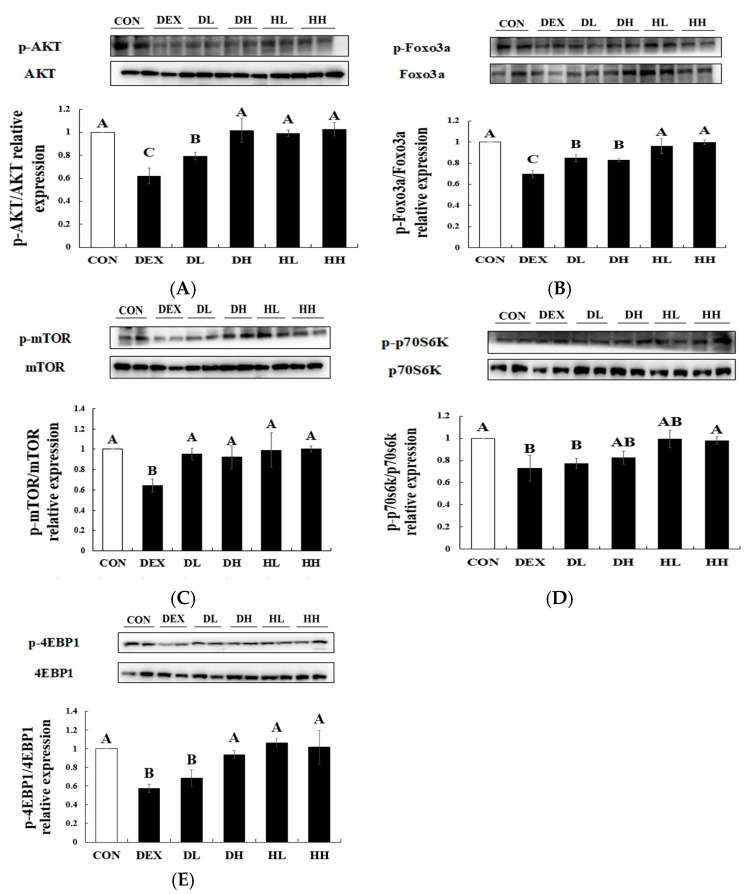
Effect of A4 < 3kDa on the AKT/mTOR/FoxO3a signaling pathway in dexamethasone-treated mice. (**A**) AKT phosphorylation. (**B**) Foxo3a phosphorylation. (**C**) mTOR phosphorylation. (**D**) p70S6K phosphorylation. (**E**) 4EBP1 phosphorylation. CON = normal group; DEX = dexamethasone-treated group; DL = A4 < 3kDa (200 mg/kg BW)-administrated and dexamethasone-treated group; DH = A4 < 3kDa (500 mg/kg BW)-administrated and dexamethasone-treated group; HL = A4 < 3kDa (200 mg/kg BW)-administrated group; HH = A4 < 3kDa (500 mg/kg BW)-administrated group. ^A–C^ Values of bar with different superscripts among treatments differ significantly at *p* < 0.05.

To assess whether A4 < 3kDa altered the molecular mechanism associated with muscle atrophy, protein levels of AKT, FoxO3a, mTOR, p70S6K, and 4EBP1 in GA muscle were examined (Figure 5). The original images of the protein bands and β-actin are provided in the Supplementary Materials. Due to the high molecular weight of mTOR, its blot was performed separately from the other proteins. The phosphorylation levels of AKT, FoxO3a, mTOR, p70S6K, and 4EBP1 significantly declined in the DEX group compared to the CON group, and this decrease was markedly accentuated by treatment with 200 mg/kg BW of A4 < 3kDa. The administration of A4 < 3kDa resulted in notable increases in the phosphorylation of AKT, FoxO3a, and mTOR by 63.77%, 18.97%, and 47.67%, respectively. Additionally, the phosphorylation of p70S6K and 4EBP1, downstream effectors of mTOR, showed enhancements of 13.05% and 63.46%, respectively, at a dose of 500 mg/kg BW of A4 < 3kDa.

Lee et al. stressed the importance of nutrients in food that are essential for muscle tissue metabolism and play pivotal roles in muscle differentiation and skeletal muscle function. They specifically underscored the role of proteins, particularly branched-chain amino acids (BCAAs) such as leucine, isoleucine, and valine, which are key in inhibiting the activation of FoxO3a [39]. Notably, horse meat has been found to contain higher levels of BCAAs compared to pork and beef [16,40]. Among the BCAAs, leucine is known to activate mTOR while concurrently inhibiting AMP-activated protein kinase (AMPK), a suppressor of protein synthesis [39]. Horse meat is noted for having approximately 2.07 times and 1.25 times more leucine than beef and pork, respectively [16,40]. Given these attributes, the increased phosphorylation of AKT, mTOR, FoxO3a, 4EBP1, and p70S6K observed upon treating A4 < 3kDa may arise from its high BCAA content, particularly leucine. However, since leucine or leucine-containing peptides were not directly identified or analyzed in A4 < 3kDa, and the current discussion is based on the previous literature, further studies are needed to identify the specific amino acids or peptides responsible for the observed effects and to determine whether similar outcomes can be replicated using isolated components. There are other previous studies involving hydrolysates with significant protein concentrations. Jeon and Choung produced two types of oyster hydrolysates: one hydrolyzed with Protamex^®^ and Neutrase^®^ (TGPN) and another hydrolyzed with the same enzymes following a 5 min blanching (PNY) [26]. Their findings indicated that the phosphorylation levels of AKT, mTOR, p70S6k, and 4EBP1 increased by approximately 85.71%, 42.86%, 71.43%, and 66.67%, respectively, following the administration of 400 mg/kg BW of TGPN. Similarly, when 400 mg/kg BW of PNY was administered to immobilized mice, phosphorylation levels rose to 78.57%, 42.86%, 45.71%, and 66.67%. Furthermore, a study by Shin et al. produced whey protein hydrolysate using Alcalase^®^, Protamex^®^, and Flavourzyme^®^, followed by filtering the unhydrolyzed proteins through a 1 μm filter paper [41] The results revealed an increase in phosphorylation of AKT, mTOR, 4EBP1, and p70S6K by approximately 18.75%, 26.67%, 24%, and 25%, respectively, after administering whey protein hydrolysate at a concentration of 400 mg/kg BW. From these findings, the enhanced phosphorylation effect of A4 < 3kDa could be linked to the distinctive characteristics of horse meat, renowned for its high protein content.

### 3.4. mRNA Expression Levels of Ubiquitin Proteasomes Associated with Muscle Atrophy in Dexamethasone-Treated Mice

**Figure 6:**
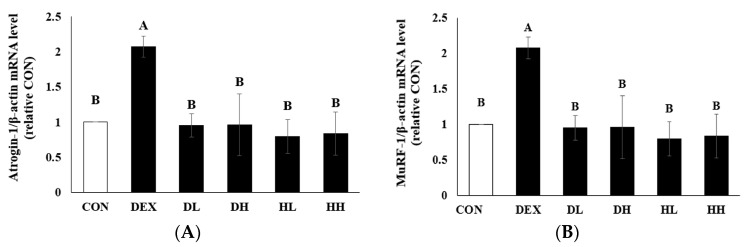
Inhibitory effect of A4 < 3kDa on mRNA expression level of Atrogin-1 and MuRF-1 in dexamethasone-treated mice. (**A**) Relative mRNA expression of Atrogin-1. (**B**) Relative mRNA expression of MuRF-1. CON = normal group; DEX = dexamethasone-treated group; DL = A4 < 3kDa (200 mg/kg BW)-administrated and dexamethasone-treated group; DH = A4 < 3kDa (500 mg/kg BW)-administrated and dexamethasone-treated group; HL = A4 < 3kDa (200 mg/kg BW)-administrated group; HH = A4 < 3kDa (500 mg/kg BW)-administrated group. ^A,B^ Values of bars with different superscripts among treatments differ significantly at *p* < 0.05.

Ubiquitination regulates protein degradation in various models of skeletal muscle atrophy, where an increase in ubiquitin-proteasome activation is frequently observed [42]. Subsequently, the ubiquitinated substrates are recognized and degraded by the proteasome. Recently, Atrogin-1 and MuRF-1 have been identified as primary contributors to skeletal muscle atrophy, possessing substrate recognition sites and functioning as ligases that facilitate protein ubiquitination [43]. Consequently, we explored the capacity of A4 < 3kDa to mitigate proteasomal protein degradation (Figure 6).

In the DEX group, the mRNA expression levels of Atrogin-1 and MuRF-1 increased 2.03-fold and 2.07-fold, respectively, due to decreased phosphorylation of FoxO3a. Treatment with 200 mg/kg BW of A4 < 3kDa significantly reduced the mRNA expression of Atrogin-1 and MuRF-1 by up to 54.49% and 54.05%, respectively, ultimately reaching levels comparable to those observed in the CON group.

Moreover, other studies using extracts or hydrolysates from natural sources reported inhibitory effects similar to those demonstrated by A4 < 3kDa. Lee et al. found that administering 200 mg/kg of ethanol extract from A. *japonica* decreased the mRNA expression level of MuRF-1 by about 66.29% and Atrogin-1 by 34.62% in the GA muscle of dexamethasone-treated C57BL/6 mice [44]. Additionally, a study by Han et al. revealed that administering a mix of 800 mg/kg BW of whey protein hydrolysate produced using Alcalase^®^, Flavourzyme^®^, and Protamex^®^, combined with 100 mg/kg BW of water extract from Panax ginseng berry to immobilized C57BL/6 mice for 2 weeks, reduced the mRNA levels of Atrogin-1 and MuRF-1 by approximately 62.5% and 54.17%, respectively. Although the compositions of A4 < 3kDa and the whey protein hydrolysate combined with ginseng extract were not identified, both studies employed Alcalase^®^ to enzymatically process natural substrates and observed protective effects against muscle atrophy. Therefore, the previous study was cited as a comparative reference to contextualize the efficacy of low-molecular-weight hydrolysates derived from natural sources [45].

The observed inhibitory effects of A4 < 3kDa are linked to the increased weight and muscle fiber CSA of the GA muscle. The expression of Atrogin-1 and MuRF-1 was reduced by IGF-1, a well-studied factor known for its role in protein synthesis. Notably, in the case of Atrogin-1, IGF-1 can rapidly decrease its expression level by approximately 30% within 1 h [46]. Previous research indicated that mice overexpressing IGF-1 in muscle displayed increased muscle mass, fiber CSA, and isometric force [47]. Conversely, mice with muscle-specific loss of the IGF-1 receptor exhibited reduced whole-body and muscle mass, as well as decreased muscle fiber CSA [48]. Given that IGF-1 also activates mTOR through AKT [49], the treatment with A4 < 3kDa may up-regulate IGF-1 activation, thereby attenuating muscle atrophy in dexamethasone-treated C57BL/6 mice. While IGF-1 was not directly assessed, the observed upregulation of AKT/mTOR signaling and downregulation of Atrogin-1 and MuRF-1 may indirectly suggest enhanced IGF-1 pathway activity following the A4 < 3kDa treatment. This hypothesis regarding the relationship between A4 < 3kDa and IGF-1 signaling should be validated in future studies to determine whether A4 < 3kDa directly binds to the IGF-1 receptor or promotes the expression of IGF-1 itself.

Compared to the CON group, no significant changes in the mRNA expression levels of Atrogin-1 and MuRF-1 were observed in the HL and HH groups. Two potential explanations may account for this observation. First, the anti-atrophic effects of A4 < 3kDa may be mediated indirectly through its antioxidative and anti-inflammatory properties, as evidenced by the reduction in intracellular reactive oxygen species (ROS) levels and the downregulation of pro-inflammatory cytokine mRNA expression, as previously reported [21]. Second, the absence of significant changes may be due to the dose and duration of A4 < 3kDa administration. For instance, in a previous study, 8-week-old male C57BL/6 wild-type mice fed a high-protein diet (providing 60% of total energy from protein) for 12 weeks showed a modest increase in body fat mass (from ~10% to 14%) and a substantial increase in lean body mass (from ~84% to 90%), indicating enhanced muscle accretion. Based on average food intake data from previous studies, mice consume approximately 28.93 mg of protein per week [50]. Considering that horse meat contains approximately 24.5% protein, administration of A4 < 3kDa at a dose of 500 mg/kg is estimated to supply about 20.58 mg of protein per week, which may be insufficient to elicit measurable hypertrophic effects under normal conditions. Therefore, further studies are warranted to elucidate the dose- and time-dependent effects of A4 < 3kDa on muscle mass and atrophy-related gene expression in both normal and catabolic conditions.

## 4. Conclusions

The current study demonstrated that horse meat hydrolysate, A4 < 3kDa, protected against muscle atrophy induced by increased glucocorticoid levels. A4 < 3kDa elevated the body weight and increased the mass of hind-limb muscle tissues (quadriceps, tibialis anterior, and gastrocnemius) and improved grip strength in dexamethasone-treated C57BL/6 mice. These effects were associated with the enhanced phosphorylation of key elements in the protein synthesis pathway, including AKT, mTOR, p70S6k, and 4EBP1, as well as FoxO3a, which plays a role in protein degradation. Concurrently, proteolytic markers Atrogin-1 and MuRF-1 decreased following treatment with A4 < 3kDa. While animal-derived foods are commonly recognized as sources of protein, earlier studies have largely utilized extracts from herbs and brown algae or chemical compounds derived from plants, focusing on their anti-inflammatory or antioxidant properties. However, the importance of protein and essential amino acid supplementation as a vital nutritional strategy to combat muscle atrophy highlights the significance of consuming foods rich in animal proteins. Nonetheless, studies exploring animal-derived materials to examine protective effects against muscle atrophy remain limited. Hence, A4 < 3kDa may serve as a significant component in the development of functional foods.

However, several key challenges and future directions must be considered before practical application. First, the exact bioactive components within A4 < 3kDa responsible for the observed effects remain unidentified. Although it is presumed to contain a mixture of bioactive oligopeptides and amino acids, as reported in previous studies employing similar preparation methods [51,52,53], for broader application and mechanistic understanding, the identification of the specific peptide sequences responsible for the observed effects is warranted. Second, while A4 < 3kDa showed protective effects against dexamethasone-induced atrophy, its direct role in anabolic signaling and lack of hypertrophic effect under normal conditions remain unclear. Further studies are needed to clarify its molecular targets and efficacy across different physiological states, using naturally aged rodents and immobilized mice. Third, since no clinical trials have yet evaluated the efficacy of this product in preventing muscle mass loss following dexamethasone administration, an extrapolation of the current findings to humans should be approached with caution. Rigorous validation through additional preclinical and clinical studies is essential. Furthermore, challenges such as formulation stability, sensory characteristics, and compatibility with various food matrices must be addressed to facilitate the development of consumer-acceptable functional food applications. Lastly, further research using alternative animal-derived protein sources is warranted to more comprehensively evaluate the anti-atrophic potential of A4 < 3kDa.

## Figures and Tables

**Table 1:** Primer sequences and annealing temperatures for qRT-PCR analysis.

Gene Symbol	Primer Sequences		Annealing Temperature
Atrogin-1	Forward	5′-CGACCTGCCTGTGTGCTTA-3′	60 °C
	Reverse	5′-GTCCACGTCAGCAATCATCC-3′	
MuRF-1	Forward	5′-TGCCTACTTGCTCCTTGTGC-3′	
	Reverse	5′-CACCAGCATGGAGATGCAGT-3′	
β-actin	Forward	5′-AGACTTCGAGCAGGAGATGG-3′	
	Reverse	5′-ACCGCTCGTTGCCAATAGT-3′	

**Table 2:** Effect of A4 < 3kDa on body weight of C57BL/6 mice.

Experimental Days	Body Weight (g)					
	CON	DEX	DL	DH	HL	HH
1	21.60 ± 0.37	21.87 ± 0.47	21.47 ± 0.50	21.10 ± 0.42	20.92 ± 0.27	21.63 ± 0.25
7	22.10 ± 0.40	22.40 ± 0.72	21.83 ± 0.42	21.60 ± 0.38	21.77 ± 0.30	22.25 ± 0.48
14	22.25 ± 0.36	22.28 ± 0.69	22.10 ± 0.40	21.82 ± 0.31	21.89 ± 0.27	22.25 ± 0.48
21	22.77 ± 0.36	22.73 ± 0.59	22.55 ± 0.38	22.35 ± 0.43	22.37 ± 0.38	22.52 ± 0.68
28	23.68 ± 0.41 A	21.90 ± 0.49 B	23.00 ± 0.41 A	22.97 ± 0.59 A	23.05 ± 0.45 A	23.10 ± 0.50 A
35	24.58 ± 0.70 A	22.20 ± 0.36 C	23.32 ± 0.33 BC	23.23 ± 0.62 BC	23.58 ± 0.55 AB	23.62 ± 0.71 AB

CON = normal group; DEX = dexamethasone-treated group; DL = A4 < 3kDa (200 mg/kg BW)-administrated and dexamethasone-treated group; DH = A4 < 3kDa (500 mg/kg BW)-administrated and dexamethasone-treated group; HL = A4 < 3kDa (200 mg/kg BW)-administrated group; HH = A4 < 3kDa (500 mg/kg BW)-administrated group. Values are expressed as mean ± SD, A–C: Means within a row with different superscripts differ significantly at *p* < 0.05.

## Data Availability

The authors state that all data supporting the conclusions of this study are included in the article. Additional raw and processed data can be provided by the corresponding author upon reasonable request.

## Supplementary Materials

The following supporting information can be downloaded at: https://www.mdpi.com/article/10.3390/cells14141050/s1, Figure S1: MALDI-TOF MS profile of A4 < 3kDa.; Figure S2. Original western blot figure for Figure 5A,B,D,E. Left to right (two samples per group): CON, DEX, DL, DH, HL, HH. (A) AKT. (B) p-AKT. (C) Foxo3a. (D) p-Foxo3a. (E) P70S6K. (F) P70S6K. (G) 4EBP1. (H) p-4EBP1. (I) β-actin.; Figure S3. Original western blot figure for Figure 5C. Left to right (two samples per group): CON, DEX, DL, DH, HL, HH. (A) mTOR. (B) p-mTOR. (C) β-actin.
