# Supplementary material for: Horse Meat Hydrolysate Ameliorates Dexamethasone-Induced Muscle Atrophy in C57BL/6 Mice via the AKT/FoxO3a/mTOR Pathway
Source: Cells. 2025 Jul 9;14(14):1050. doi: 10.3390/cells14141050 (PMC12293372; doi:10.3390/cells14141050)
Supplement: Supplementary file 1 [file cells-14-01050-s001.zip › cells-3714790-supplementary.pdf]

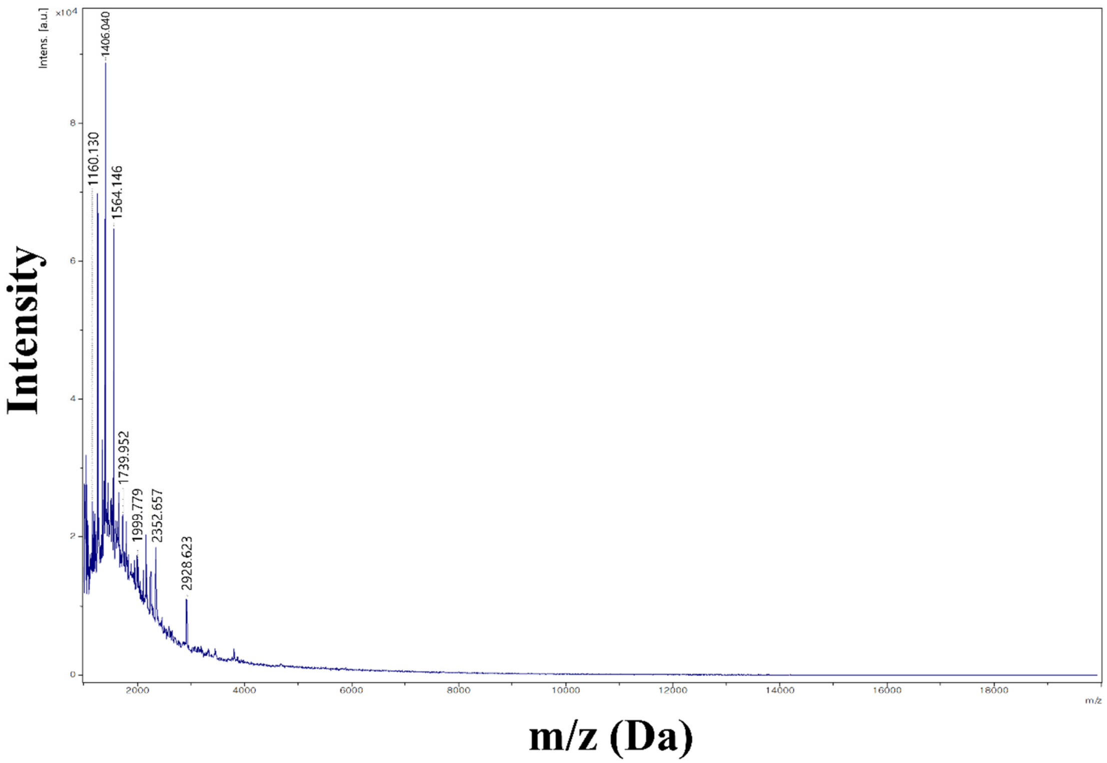

Figure S1. MALDI-TOF MS profile of A4<3kDa.

(A)

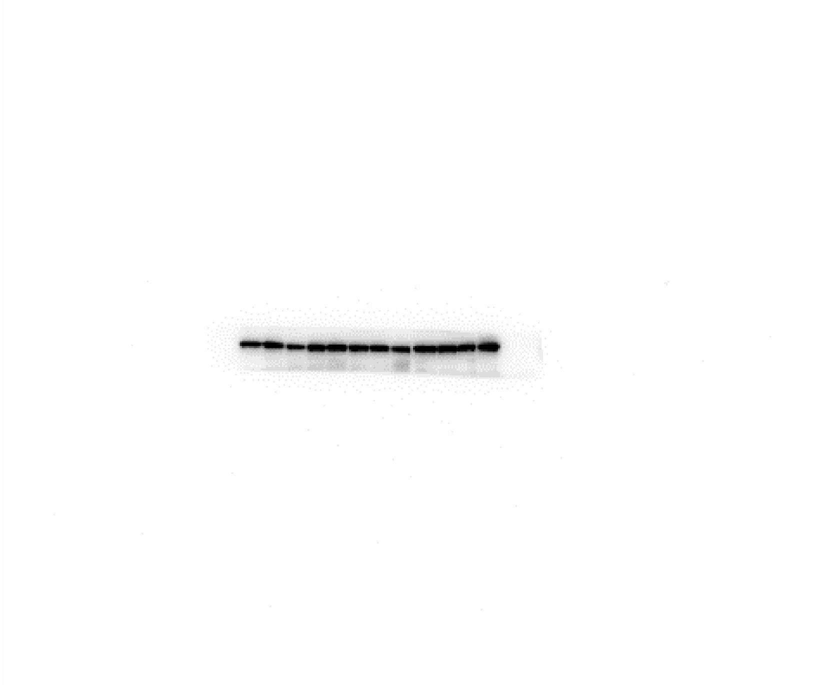

(B)

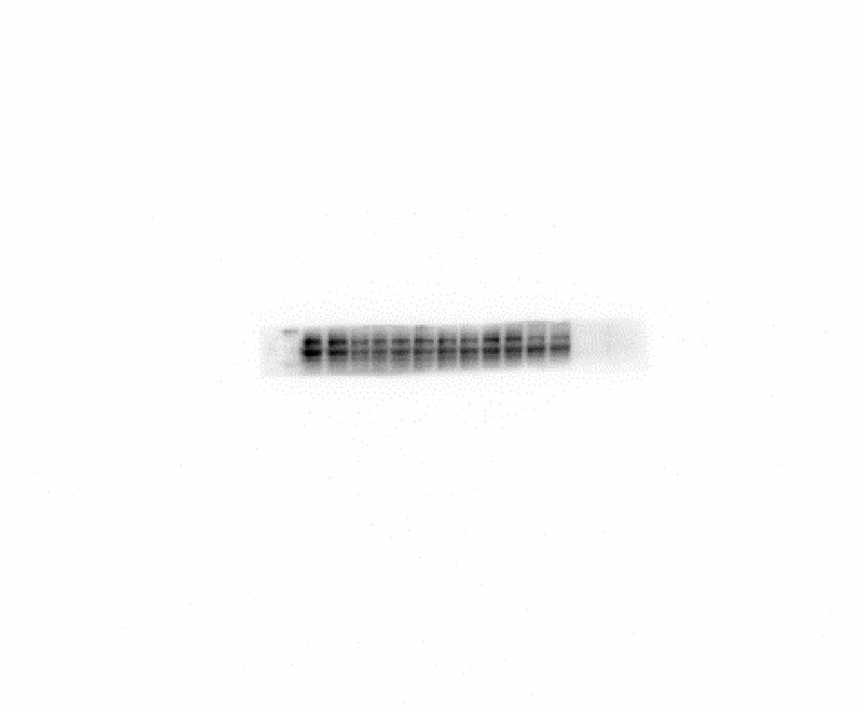

(C)

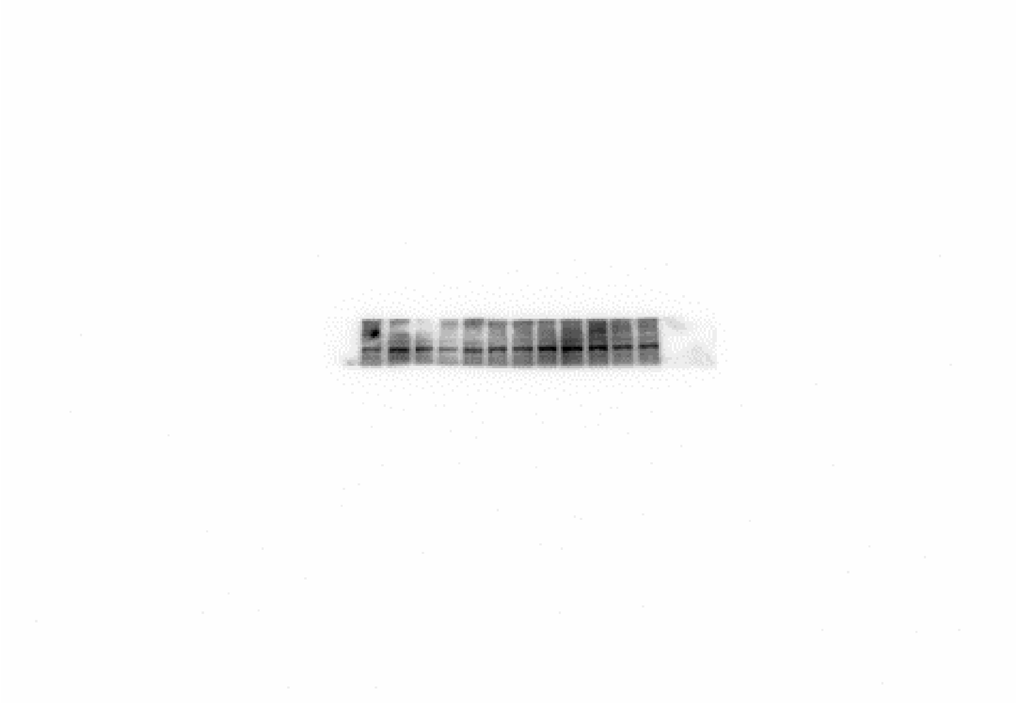

(D)

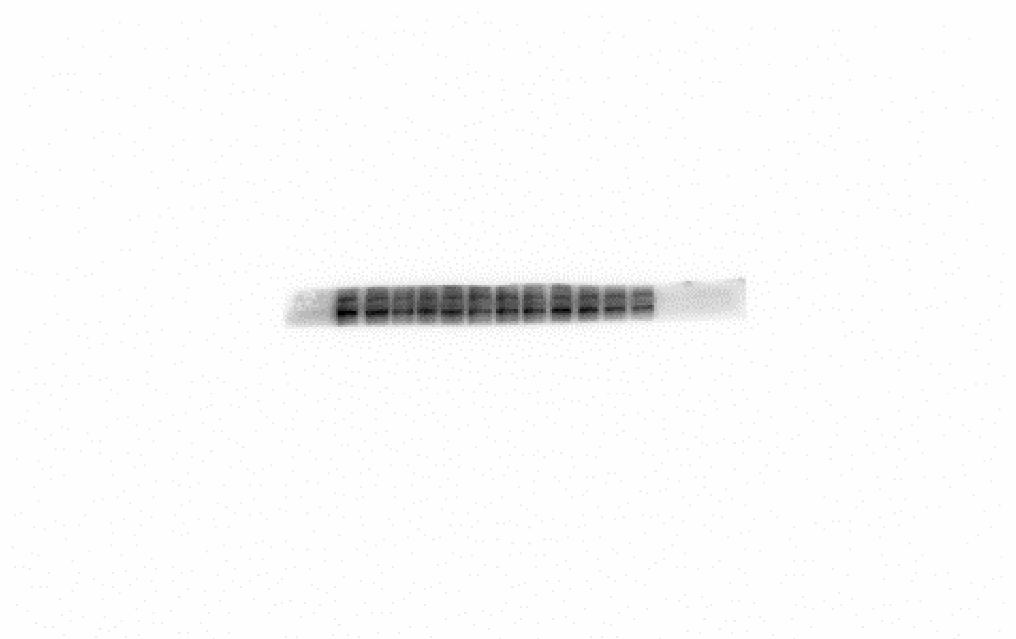

(E)

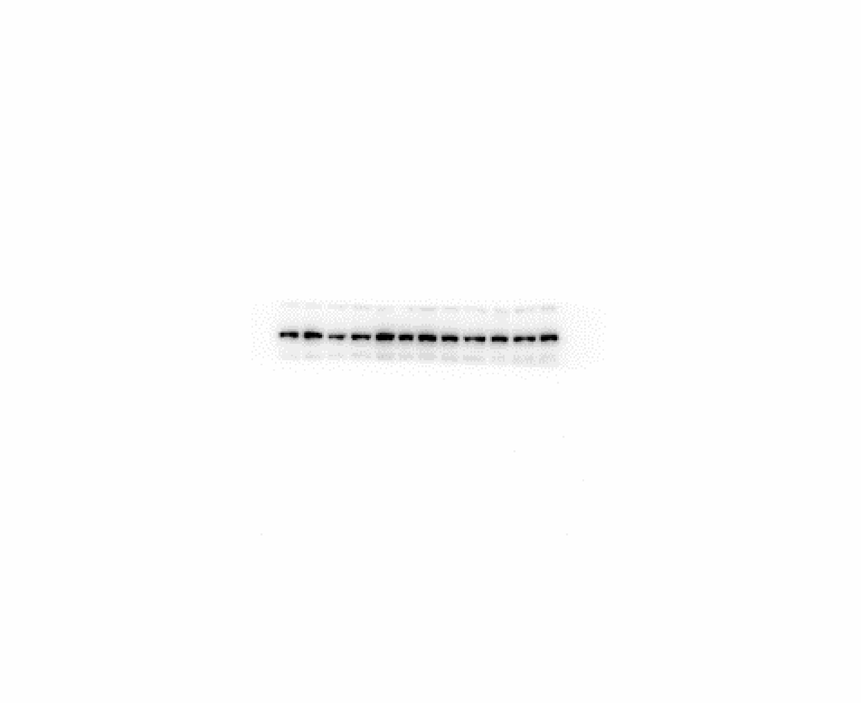

(F)

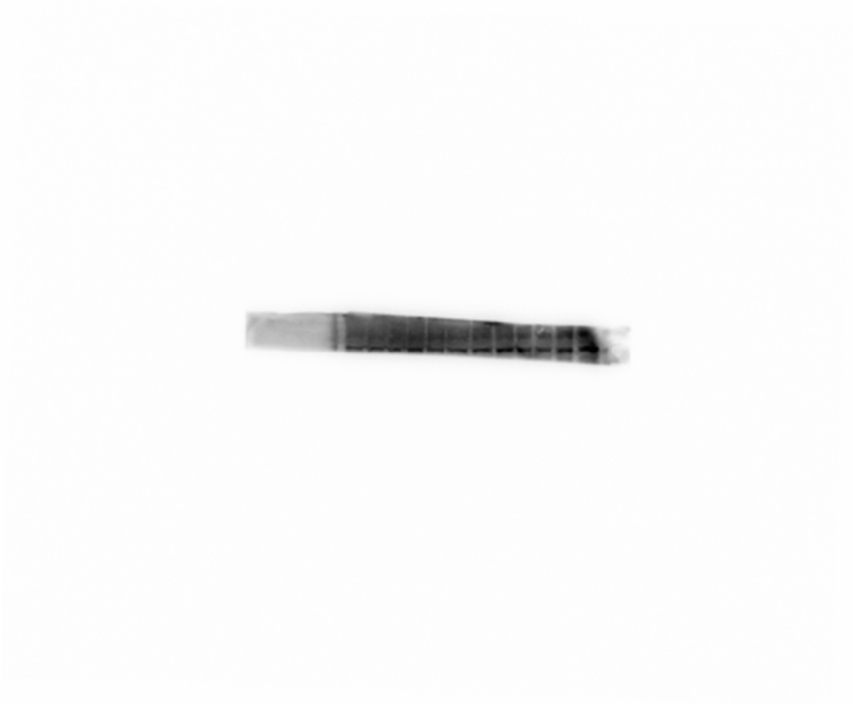

(G)

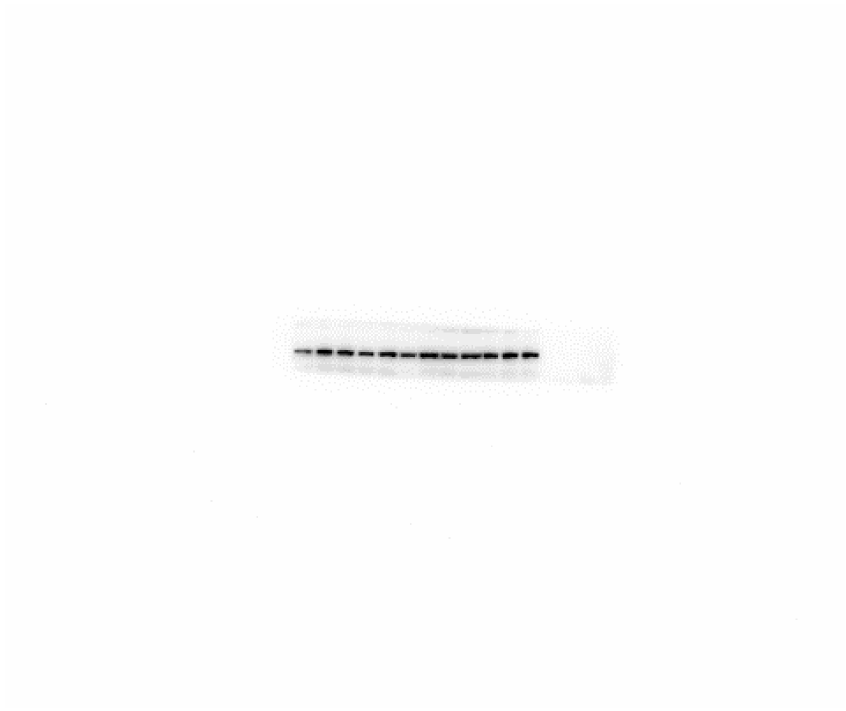

(H)

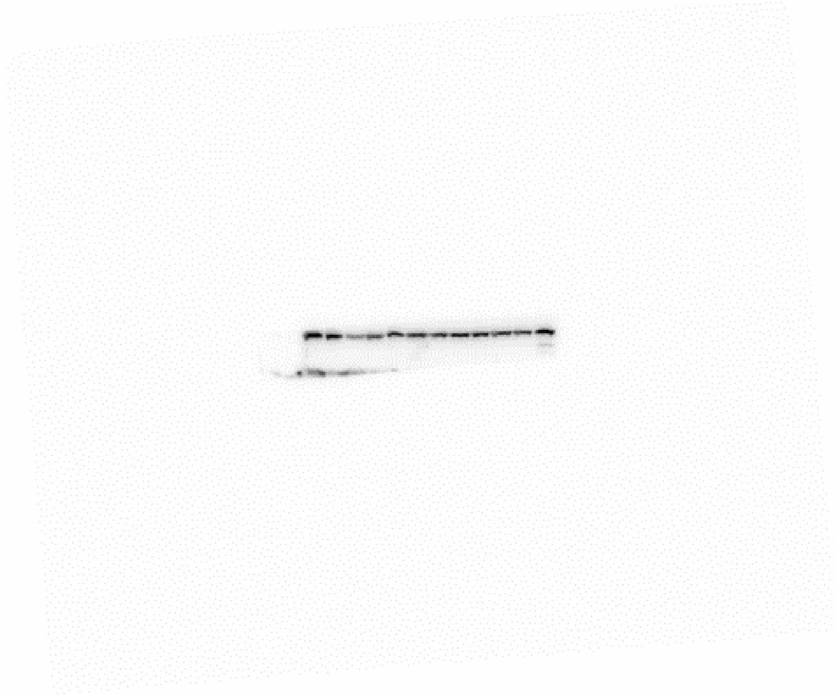

(I)

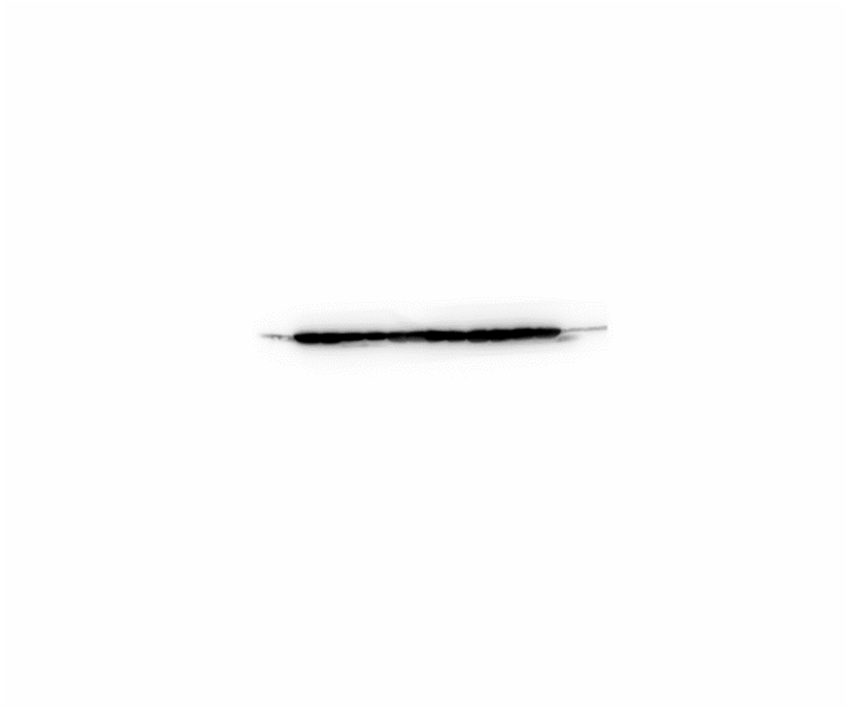

Figure S2. Original western blot figure for Figure 5A,5B, 5D and 5E. Left to right (two samples per group): CON, DEX, DL, DH, HL, HH. (**A**) AKT. (**B**) p- AKT. (**C**) Foxo3a. (**D**) p-Foxo3a. (**E**) P70S6K. (**F**) P70S6K. (**G**) 4EBP1. (**H**) p-4EBP1. (**I**) β-actin.

(A)

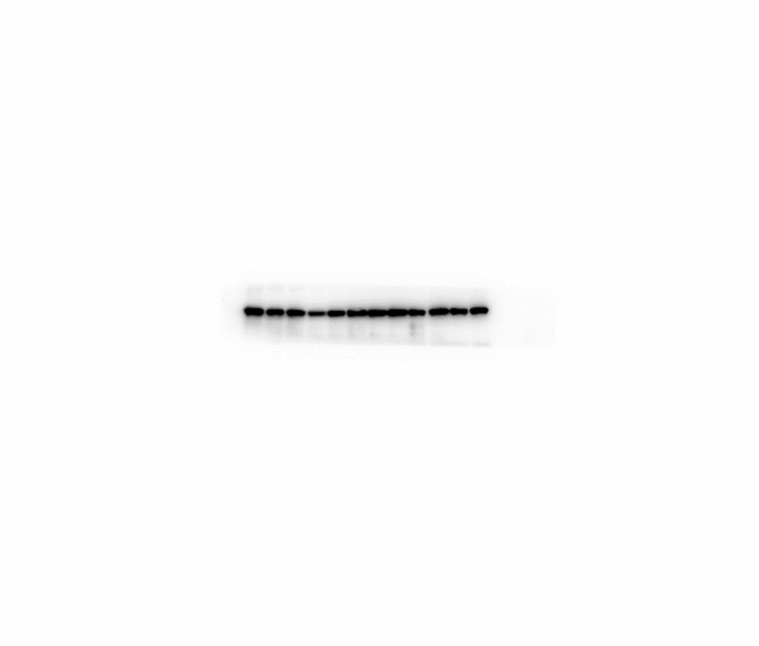

(B)

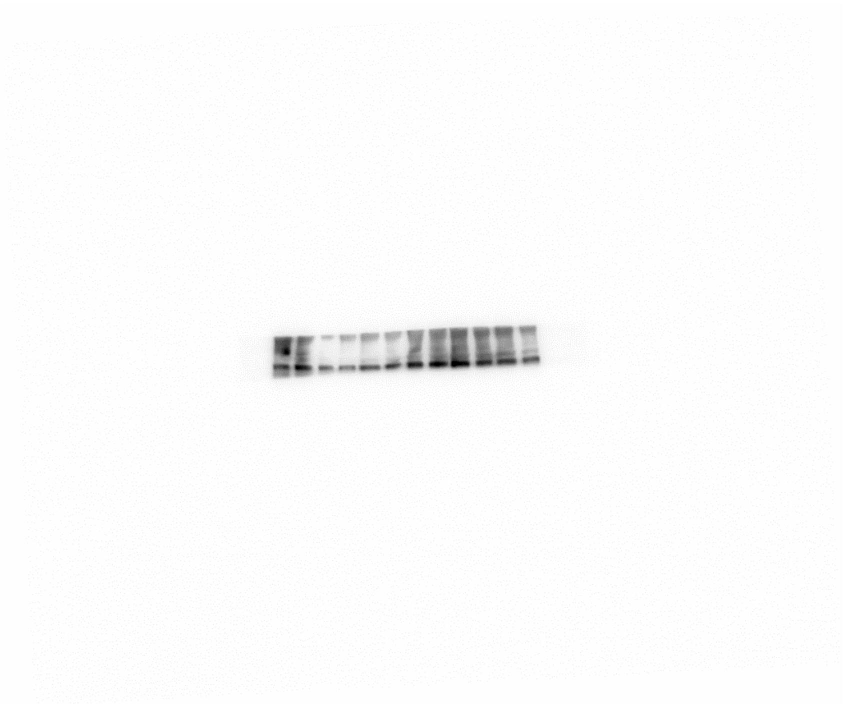

(C)

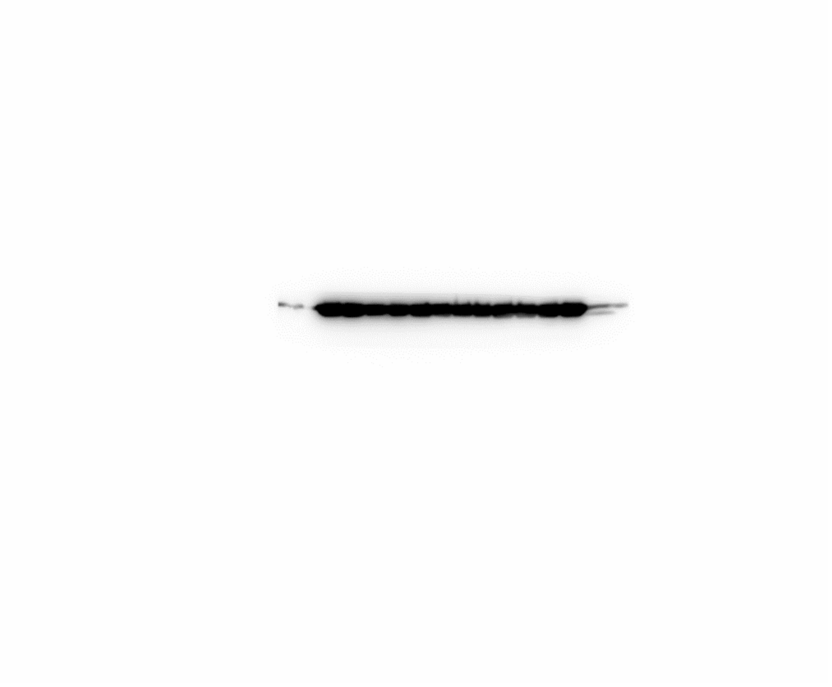

Figure S3. Original western blot figure for Figure 5C. Left to right (two samples per group): CON, DEX, DL, DH, HL, HH. (A) mTOR. (B) p-mTOR. (C) β-actin.
